# Gait-Based Diplegia Classification Using LSMT Networks

**DOI:** 10.1155/2019/3796898

**Published:** 2019-01-17

**Authors:** Alberto Ferrari, Luca Bergamini, Giorgio Guerzoni, Simone Calderara, Nicola Bicocchi, Giorgio Vitetta, Corrado Borghi, Rita Neviani, Adriano Ferrari

**Affiliations:** ^1^Department of Electrical, Electronic and Information Engineering Guglielmo Marconi, University of Bologna, Viale Risorgimento 2, 40136 Bologna, Italy; ^2^Department of Engineering Enzo Ferrari, University of Modena and Reggio Emilia, Via Vivarelli 10, 41125 Modena, Italy; ^3^LAMBDA -Laboratorio Analisi del Movimento del Bambino Dis-Abile, Azienda Ospedaliera Arcispedale S. Maria Nuova and University of Modena and Reggio Emilia, Reggio Emilia, Italy; ^4^Department of Neuroscience, University of Modena and Reggio Emilia, Reggio Emilia, Italy

## Abstract

Diplegia is a specific subcategory of the wide spectrum of motion disorders gathered under the name of cerebral palsy. Recent works proposed to use gait analysis for diplegia classification paving the way for automated analysis. A clinically established gait-based classification system divides diplegic patients into 4 main forms, each one associated with a peculiar walking pattern. In this work, we apply two different deep learning techniques, namely, *multilayer perceptron* and *recurrent neural networks*, to automatically classify children into the 4 clinical forms. For the analysis, we used a dataset comprising gait data of 174 patients collected by means of an optoelectronic system. The measurements describing walking patterns have been processed to extract 27 angular parameters and then used to train both kinds of neural networks. Classification results are comparable with those provided by experts in 3 out of 4 forms.

## 1. Introduction

Cerebral palsy (CP) is a group of permanent movement disorders appearing in early childhood. It is widely considered as a neurodevelopmental disorder because it affects the most sensitive period of the development of human beings. Signs and symptoms of CP vary among people. Typical symptoms are poor coordination, stiff or weak muscles, and disturbances affecting perception, vision, hearing, swallowing, and speaking. Even if symptoms may get more noticeable over first few years of life, underlying impairments do not worsen over time.

Treating CP usually requires a multidisciplinary effort by neurologists, rehabilitation specialists, and therapists for identifying the best clinical solutions for each patient. Available treatments include physical therapy, muscle relaxants, and functional surgery, and their adoption depends on the CP class (or more properly *form*) patients belong to. To separate children in forms has the scope of assisting in diagnosis formulation, clinical decision-making, and communication. A form can be conceived as a framework, shared by different clinicians, able to quickly convey a clinical snapshot of a child by cross-referring to histories of other patients with similar motor impairments. Therefore, a good classification system separates patients into clusters characterized by sharing comparable prognosis, thus easing choice of treatments and communication of the expectations on the autonomy level during child growth [1]. Given the variety of movement disorders comprised under the CP umbrella term, multiple classification criteria have been proposed. On the one hand, when the identification of muscle tone anomalies together with the type of prevailing neurological symptom are considered, patients can be classified as (i) *spastic*, suffering from constant muscles tightness and stiffness; (ii) *dyskinetic*, associated with inability to control involuntary movements; and (iii) *ataxic*, associated with shakiness and lack of coordination.

On the other hand, when the somatic location of prevailing neurological symptom is observed, CP can be distinguished as (i) *tetraplegia* affecting all four limbs; (ii) *diplegia*, with lower limbs more compromised than upper ones; (iii) *hemiplegia*, affecting only one side of the body; and (iv) *monoplegia*, influencing a single limb.

Besides the above cited classification criteria, the Gross Motor Function Classification System (GMFCS [2]) is one of the most used and useful tool to assess the level of autonomy of CP patients by addressing their gross motor competence. Yet, it does not offer any element to infer prognosis neither to assist clinical decision-making.

In the group of diplegia, with the aim of realizing an effective classification system, Ferrari et al. proposed moving the focus of clinical observation on central (top-down) components of motor organization [3, 4]. Here, top-down components are intended as the main features of motor organization spontaneously performed by individuals in order to find and maintain a safe, stable, and efficient gait. In particular, these are (i) support reaction to body weight, i.e., pattern adopted to find and maintain balance, (ii) static and dynamic fixation mechanisms, i.e., modality of use of canes, and position and swing of upper limbs, (iii) step-by-step righting reactions, i.e., trunk and pelvis pendulum and translation in and out of sagittal plane, (iv) mechanisms of progression, i.e., choice of fulcra upon which to pivot the body, and (v) ability to correctly integrate proprioceptive and perceptive information [3, 5].

Based on this criteria, Ferrari et al. have recognized four different forms of motor organization separated in terms of which and how much motor repertoire is accessible to patients (Table 1). In particular, shifting from form I to IV, children show a motor repertoire increasingly rich and adaptive to environment. Form IV is characterized by a high functioning central nervous system that allows for coping even with severe peripheral deficits and executing complex motor activities. Instead, form I presents a low functioning control system that, even without major peripheral dysfunctions, limits motor performance [3].

This classification has proved to be effective in easing clinical assessments, planning treatments, and measuring their effectiveness [3, 5, 6]. In particular, it has been proved effective in assisting surgical planning [6] and has been validated on a group of 67 children suffering from diplegia [7]. The results obtained were encouraging and evidenced that GMFCS levels increased from form I to IV, enhancing the idea that, inside the umbrella term diplegia, there are different groups of patients [7]. Furthermore, in a related work involving a group of 50 patients, it was demonstrated how this classification can be learned rapidly and present an excellent interobserver reliability [8]. Generally speaking, this latter study has evidenced that (i) forms I and IV are the most easily identifiable; (ii) form III can be identified by observing the frontal swing of trunk and upper limbs; and (iii) the main feature of form II, knee flexion in midstance, is less evident.

Our research work relies on the above findings and investigates the implementation of a reliable and automatic system capable of identifying the four forms of spastic diplegia as defined in [3, 4]. The proposed system is based on deep learning techniques and, consequently, does not involve the extraction of domain-specific features for classification. The aim is to provide a contribution to the validation of this classification by demonstrating that the four clinical forms are artificially recognizable in the space of quantitative gait data. Besides, such system may endow rehabilitation centres with an accurate tool for quickly confirming diagnosis and for clinical decision-making.

The remaining part of the manuscript is organized as follows. In Section 2, related work in the field of diplegia classification is illustrated. In Section 3, gait analysis is introduced, and sensors and methodologies used for collecting gait data are described. In Sections 4 and 5, the use of *multilayer perceptron* (MLP) and *recurrent neural networks* (RNNs) is illustrated. Results are discussed in Section 6.  Section 7 concludes the paper.

## 2. Related Work

Recent research on automatic classification of CP has been mostly aimed at discerning between CP patients and asymptomatic subjects instead of classifying different forms of the disorder. Furthermore, as far as we know, previous work mostly used *support vector machines* [9, 10] and classical *neural networks* [11–13].

The use of SVM as binary classification for the detection of spastic diplegia has been investigated in [9]. The proposed classifier has been trained on a dataset of gaits referring to 88 children affected by spastic diplegia together with a control group of 68 typically developed children. A six-camera optoelectronic system has been employed for data acquisition, and raw data have been processed to extract three distinct features (namely, stride length, cadence, and leg length). The accuracy of classification from SVM has been assessed for different feature sets and kernel functions. In practice, the best performance results have been obtained employing stride length and cadence (normalized on the basis of leg length and age) and selecting a radial basis function as kernel; more specifically, an overall accuracy of 96.8% has been achieved adopting a 10-fold cross validation. SVM methods have also been employed in [10] to solve the problem of spastic hemiplegia classification. In this case, the binary classificator has been trained using a rich dataset, referring to more than 900 trials divided into different classes corresponding to different pathologies. The data have been acquired with an optoelectronic system and then processed to estimate joint kinematics.

A specific type of artificial neural network, known a *self-organizing map* (SOM), has been exploited in [11] to learn an abstract representation of a normal gait pattern from a dataset of 129 gait cycles acquired from 18 typically developed subjects. The method developed for classification is based on the idea that the degree of motor disorder can be identified by evaluating the *quantisation error* (QE) of the differences between normal and abnormal gait patterns. In this case, the features used for training the SOM and computing the QE were three-dimensional joint angles, moments, and powers.

### 2.1. A Bayesian Network (BN)

Bayesian network has been employed in [12] to identify patterns underlying gait deviations on the basis of gait data acquired from 139 CP patients. This approach has offered the relevant advantage of incorporating clinical expertise as prior knowledge in the classification method; this has eased the analysis of available data and has helped identifying clinically relevant relationships. The prior knowledge of clinical patterns has been introduced through various iterations, involving a multidisciplinary team of experts in fields of 3D gait analysis and CP. It is also worth mentioning that (i) the BN output consisted of the probabilities of suffering from a specific gait deviation pattern instead of a single label referring to the most likely gait pattern; (ii) the trained BN achieved an average accuracy rate of 88.4% with respect to a gold standard represented by clinical experts.

### 2.2. Artificial Neural Networks (ANNs)

ANNs have been shown in [13] to outperform previous logistic regression approaches in terms of accuracy. The ANN architecture described here consisted of one single hidden layer with just 12 neurons, enough to obtain a better score in the *receiver operating characteristic* (ROC) plots. The processed data included not only simple personal information of patients (e.g., age and gender) but also data about their heart rate variability (correlated with the neurodevelopment outcome during early life). The ANN has been tested on a dataset referring to 35 infants diagnosed with central coordination disturbances, against a control group of 37 asymptomatic subjects.

### 2.3. Principal Component Analysis (PCA)

PCA has been applied to gait features extracted from 40 subjects (more precisely, 20 typically developed subjects and 20 subjects with spastic diplegia) [14]. This allowed to identify three principal components accounting for 61% of total variability. Moreover, a graphical representation of these components has been exploited as a classification tool since a significant distance between typically developed and diplegic subjects was found in the plots obtained.

In [1], various different studies on children affected by CP were jointly analysed. In this review study, the considered tools span from traditional systems for data analysis to more recent machine learning algorithms, such as SVM techniques and *generalized neural networks*.

To conclude, it is worth mentioning that none of the studies present in literature investigated the problem of setting up an automatic classification system based on the criteria proposed in [4].

## 3. Dataset for Gait Analysis

Gait analysis is the study of human locomotion, augmented by instrumentation for measuring biomechanics of movements and the activity of muscles. Gait analysis is typically used to assess individuals whose conditions affect their ability to walk effectively and safely.

Gait analysis usually takes place in dedicated laboratories called *motion analysis labs* (MALs). The typical equipment employed in these laboratories is listed in Table 2.

### 3.1. Data Acquisition Procedure

A set of optoelectronic markers and electromyographic sensors are applied to the patient's skin. These markers and sensors allow to acquire gait kinematics and muscular activities of a patient. The complete procedure for the acquisition of gait data by means of optoelectronic devices and force plates is described in the gait analysis protocol [15].

### 3.2. Data Preprocessing

The dataset processed in this study consists of 1121 trials (i.e., walks) involving 174 patients collected in an Italian MAL, namely, the LAMBDA (Laboratorio Analisi del Movimento del Bambino Disabile) of the hospital Arcispedale S. Maria Nuova hosted in a 12 m × 8 m room equipped with a Vicon system of 8 MX + cameras (Oxford Metrics Inc., UK). Acquired data were saved in the *coordinate* 3D (C3D) format. This study was approved by the ethics committee of the Santa-Maria-Nuova hospital on 22/01/2014. None of the participants had prior knowledge of the purpose of this study. The distribution of trials and patients over the considered four forms is shown in Table 3. It should be noted that

- Patients belonging to form 4 suffer from less severe symptoms, whereas those belonging to the first two forms (i.e., forms 1 and 2) are the most hindered
- The distribution of patients over the forms in this dataset is uneven mainly due to the uneven clinical distribution of patients along the four forms [4]

The measurements comprise 19 markers (Table 4) and were subsampled by a factor 2 reducing the sampling rate from 100 frames/sec to 50 frames/sec. This made the representation of patient movements closer to human eye perception, thus making the automatic classification input comparable with the one available to clinical experts. The remaining part of data preprocessing consisted of the following tasks:

1. Identifying the average period *T* of the sequence of steps for each patient
2. Selecting the data associated with an integer number of steps
3. Transforming position information into angular information
4. Transforming the available data from time to frequency domain and organizing them in sequences
5. Partitioning the obtained dataset in a training set and in a test set

Regarding task 1, foot strikes and toe-offs were visually identified in the virtual environment of the reconstructed marker positions. Data associated with steps not completely captured at the beginning or at the end of a given trial were removed. Parameter *T* was estimated by dividing the trial duration by the number of steps it contains.

Task 3 consisted in projecting 3D coordinates of markers to the three human body's planes. Then, the projected coordinates were processed to extract 27 scalar angles in each plane (Table 5), as most of the clinical signs are strongly related to angular information [4]. Consequently, 81 angles per frame were generated.

Task 4 involved two subtasks, one concerning the frequency analysis and the other one the acquired sequences. The first subtask was inspired by [16], where it is shown that pathological gait patterns can be discerned from normal ones exploiting the Fourier analysis; the same approach was exploited here to separate different forms of diplegia. In practice, the *fast Fourier transform* (FFT) algorithm was applied to a sequence encompassing multiple (say *N*) steps. Then, one coefficient every *N* was extracted from the FFT output vector, in order to analyze only those harmonics associated with the fundamental of a single step (independently from the number of steps performed by the considered patient). It is also worth mentioning that (i) only the first 20 coefficients selected in this way were preserved (all those referring to higher frequencies were deemed not meaningful); (ii) the first selected coefficient was not normalized, whereas all the other ones (each angle and each trial) were normalized to the amplitude of the fundamental. The second subtask, instead, simply gathers sequences of 75 elements (where each element is composed of 81 3D angles), displaced by 15 elements and for a maximum of 45 sequences per trial.

Finally, in task 5, the dataset was partitioned (according to the proportion 0.75 : 0.25) patient-wise for training and testing, respectively, in each form. Trials referring to the same patient were not included in both the training and the test sets. Moreover, since patients belonging to form 1 were fewer than other forms, their number was artificially doubled by repeating every occurrence in the train set, as shown in Table 3 (information referring to augmented data is given in parentheses).

## 4. Classification with Multilayer Perceptron Network

**Figure 1:**
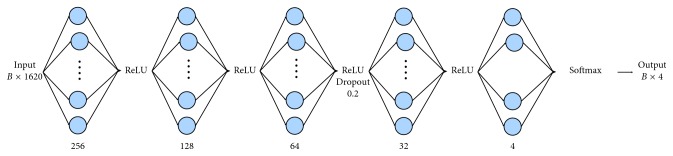
Proposed MLP network. Every cell is fully connected with the output of the previous layer through a set of weights (plus a bias) which are used to compute the new output.

We started by implementing a MLP network with a single layer (i.e., from the case of *logistic regression* with four perceptrons) and then adding further layers with a number of perceptrons evaluated on the basis of the following simple rules: (i) the first additional layer consists of 32 hidden units; (ii) any other added layer contains a number of perceptrons which is twice that of the previous layer. The best scoring network is shown in Figure 1.

Note that a more complex network usually achieves a better accuracy on the set on which it is trained at the risk, however, of overfitting it. In fact, on the one hand, the use of additional layers makes the considered network able to learn more effectively and more complicated patterns; on the other hand, the network adapts its weights to reproduce more and more accurately the training set, thus loosing its generalisation capability.

To mitigate this issue, i.e., to contrast the potential overfitting on the training set, a *dropout layer* was included [17]. This randomly turns off a portion of the perceptrons during the training phase, thus forcing it to use only part of the available weights in generating its output. Further dropout layers showed no significant improvements. Other choices made in the implementation of this classification network are summarised below.

- Categorical cross entropy loss, shown in the following equation, was employed as a *learning function*:

(1) $$\begin{array}{c} L=-\frac{1}{N*M}\overset{N}{\underset{n=0}{\sum }}\overset{M}{\underset{m=0}{\sum }}y_{t\left[\text{n,m}\right]}*\ln\left(y_{p\left[n,m\right]}\right), \end{array}$$

  where **y**_*t*[*n*, *m*]_ and **y**_*p*[*n*, *m*]_ represent the *ground truth* and the corresponding output (*prediction*), respectively, for the *m*-th form and the *n*-th sample (*M* and *N* are the overall number of forms and the overall number of samples, respectively).

(ii) *Adam gradient descent optimiser* with default parameters was adopted for the backpropagation update [18].

## 5. Classification with Recurrent Neural Network

Recurrent neural networks have been proposed for analysis of sequences exhibiting high correlation over time. In the past years, various architectures, producing different mappings between input and output sequences, have been proposed; while nowadays, the usual choice lies between *long short-term memory* (LSTM) and GRU architectures.

**Figure 2:**
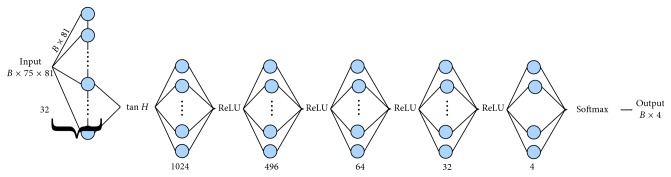
Architecture of the proposed RNN network. The network uses a many-to-many layer with *L*_1_ < *L*_2_ < 1 and a single LSTM layer.

The network we adopted exploits a single LSTM layer instead of a multilayer approach (the adoption of this strategy is motivated in the following section), as shown in Figure 2. These choices are related to the possibility of using a *backpropagation logic* and of mitigating the *overfitting*. Note that, as the number of layers in the network increases, the flow of gradient during the update phase is reduced by consecutive dot products [19] and, even if the LSTM contrasts the gradient vanishing inside each layer, this effect is still produced between layers. Another implication of the increase of complexity is the overfitting of training set due to the large number of parameters and lack of training examples [20]. We have tested this architecture making use of one or two fully connected LSTM layers over the first one; however, the simplest solution proved to be the best choice for current task in terms of generalisation capability (Section 6). Other choices we made in the implementation of this classification network are summarised below:

- The fully connected network on top of LSTM layers is appreciably deeper than the one used in the previous experiment. This is related to the adopted preprocessing step, which generates huge numbers of sequences from a single data file, allowing to introduce more layers without the risk of overfitting the train set.
- Each layer, except the LSTM one, has a ReLU activation [21]; however, a *tanh* activation was selected for the recurrent layer, since it is the one usually employed inside an LSTM cell.
- The categorical cross entropy as described in equation (1) and an Adam optimiser with default parameters was used for this network too.

## 6. Experimental Results

The performance of the proposed MLP network and RNN were assessed on a Nvidia GTX-1060 board using Keras [22]. In both cases, the stopping criterion adopted in the training process takes into account not only the loss on the training set but also resulting accuracy on both test and train sets. In our work, an architecture performing better on the test set was preferred, even if this did not achieve the best score on the training set. In practice, on the one hand, the training stage of any MLP network was completed when the loss dropped below 0.10, but when this condition was not achieved in 500 iterations on the full training set, it was always forced to an end; this stage took approximately 10 s. On the other hand, the training stage of RNNs was always ended after 15 iterations since the loss on the train set quickly dropped below 0.10. For both networks, the batch size was set to 100 elements, with reshuffle of the training dataset at the beginning of each epoch; this stage took approximately 240 s. The results show the following:

1. In the MLP case, the network with 256 hidden units in its first layer outperforms all the other MLP networks; in fact, it achieves an accuracy of about 0.587 on the test trials, whereas all other networks do not exceed 0.56.
2. In the LSTM case, overfitting occurs as training procedure proceeds over the twentieth iteration. Moreover, the one-level LSTM outperforms all other options since it achieves an accuracy of about 0.67 on test sequences, whereas other networks stop at about 0.64.

Further numerical results are listed in Tables 6 and 7, which show the accuracy scores for the top one (T1) and the top two (T2) predictions on train and test sets. Both networks were compared with a *support vector machine* with radial basis kernel function on the test set. Note that, on the one hand, the accuracy is computed on the basis of the form characterized by the highest frequency for the considered patient among all trials available for both networks; on the other hand, in the MLP case, every trial leads to a prediction used to estimate the form of the considered patient, whereas in the RNN case, every sequence contributes to the final score.

These results show the following:

1. Predictions are accurate for forms 1, 2, and 4 with just the top one score
2. Predictions are unreliable for form 3 even with the top two scores; this suggests that this form of diplegia does not have specific traits that can be recognized by the approaches proposed in this paper
3. The RNN performs better than the MLP network; in particular, the former achieves an accuracy of about 0.869 overall on patients, whereas the latter achieves an accuracy of about 0.804
4. Both methods outperform the SVM baseline

A deeper understanding of the classification problem is provided by the confusion matrices shown in Table 8. The results show that (i) a RNN provides more accurate predictions than a MLP network; (ii) the third class (class number 3 in Table 7) is the most difficult to classify, being always confused with the second one and the fourth one in the top one prediction and heavily mislabelled even in the top two, in accordance with what was clinically observed in [8]. It is worth noticing that one of the main traits of this form is a perceptual impairment [4]; this data of course does not emerge from the acquired motion data, remaining out of the knowledge of both networks.

It is worth mentioning, however, that the four forms of diplegia can partially overlap. Consequently, in practice, it can be really difficult to assign some patients to one of the four forms just using the top one prediction. In this context, RNN or MLP might also assist in identification of non-nominal cases characterized by an intermediate score between two neighbour diplegia forms [8], by assigning a probability score to each of the four forms.

As a matter of fact, the proposed classifiers perform better on opposite forms, and this confirms that a classification coherent with human observations is possible. However, as for the intermediate forms are concerned, results show that the associated behaviours are hard to identify if a small sized dataset is available; these considerations may also explain the overfitting of the train set as learning proceeds, especially for the MLP case.

Finally, it is worth mentioning that, due to the confidentiality of the processed data, the dataset has not been made public to the community; therefore, a direct comparison with other methods is not possible yet. *We commit ourselves to release the full anonymised dataset as soon as this article will be published.*

## 7. Conclusions

In this manuscript, two methods based on state-of-the-art deep learning techniques were used to solve a multiclass classification problem concerning gait data from diplegic children. More specifically, the proposed methods make use of MLP network and RNN for discriminating the 4 forms of diplegia defined in [4]. The dataset used comprises 1121 trials involving 174 patients and was preprocessed to extract angular information. Such information was then employed for training the proposed networks and for assessing their classification accuracy. Results show that RNNs slightly outperform MLP for the specific task and are in line with clinical experts in 3 out of 4 classes. These findings provide contribution to the validation of the classification proposed in [4] and promote the top-down approach to subclassify diplegia. Deep learning techniques similar to the ones here proposed might become a valuable tool in MALs as an objective reference to assist clinical professionals in the classification process.

## Figures and Tables

**Table 1:** Diplegia forms defined by Ferrari et al. [4].

Form	Main traits
Form I	Antepulsion of trunk, toe balancing. Constant support from canes
Form II	Pronounced knee flexion in midstance, loaded knee behavior, short steps
Form III	Frontal trunk swinging and use of upper limbs to keep balance, presence of dysperceptive disorders (fear of falling and of open spaces)
Form IV	Mainly a motor deficit. Increased talipes equinus at the start of walking. Difficulty to stop immediately the walking

**Table 2:** Equipment commonly employed for gait analysis.

Device	Description
Optoelectronic system	Reflecting markers attached to patients in specific anatomical landmarks allowing to acquire 3D motion of human segments
Force plate	Force plate placed on the ground to measure the intensity and direction of the reaction force to body weight
Electromyography	Skin electrodes capable of acquiring the electrical signals generated by the contraction of muscles
Video system	Cameras and other devices employed to record the movements of a patient in a walking trial

**Table 3:** Distribution of patients, trials, and sequences among the four classes (or forms) of Table 1 before and after data augmentation (in parentheses); the adopted partitioning for training and test phases is also shown.

Form	Train			Test		
	Patients	Trials	Seq.	Patients	Trials	Seq.
1	9	47 (94)	1404 (2808)	4	16	664
2	36	183	2720	13	83	1354
3	25	174	1894	9	49	712
4	58	372	3676	20	114	927

**Table 4:** Body marker IDs and their description.

Identifier	Marker position
C7	7th cervical vertebrae
LA	Left acromioclavicular joint
RA	Right acromioclavicular joint
REP	Right lateral elbow epicondyle
LEP	Left lateral elbow epicondyle
RUL	Right lateral prominence of the ulna
LUL	Left lateral prominence of the ulna
RASIS	Right anterior superior iliac spine
LASIS	Left anterior superior iliac spine
RPSIS	Right posterior superior iliac spine
LPSIS	Left posterior superior iliac spine
RGT	Right prominence of the greater trochanter
LGT	Left prominence of the greater trochanter
RLE	Right lateral knee epicondyle
LLE	Left lateral knee epicondyle
RCA	Right upper ridge of the calcaneus posterior surface
LCA	Left upper ridge of the calcaneus posterior surface
RFM	Right dorsal aspect of first metatarsal head
LFM	Left dorsal aspect of first metatarsal head

**Table 5:** Absolute 3D coordinates have been transformed into 27 three-dimensional angles, as most of the clinical signs of diplegia are strongly related to angular information.

Marker I	Marker II	Marker III
LGT	LPSIS	LLE
LLE	LGT	LCA
LCA	LLE	LFM
LEP	LA	LUL
LEP	C7	LUL
LLE	LASIS	LFM
LA	C7	LEP
RGT	RPSIS	RLE
RLE	RGT	RCA
RCA	RLE	RFM
REP	RA	RUL
REP	C7	RUL
RLE	RASIS	RFM
RA	C7	REP
LPSIS	LGT	RGT
LASIS	LGT	RGT
LPSIS	LLE	RLE
C7	LA	RA
C7	LEP	REP
RPSIS	LGT	RGT
RASIS	LGT	RGT
RPSIS	LLE	RLE
C7	LUL	RUL
LASIS	C7	LPSIS
RASIS	C7	RPSIS
LA	LASIS	RASIS
RA	LASIS	RASIS

**Table 6:** Top one accuracy scores (train set).

Form	MLP	LSTM	SVM
	Top one	Top one	Top one
1	0.98	0.97	0.98
2	0.985	0.97	0.99
3	0.8	0.96	0.95
4	0.965	0.965	0.965
Overall	0.941	0.967	0.986

**Table 7:** Accuracy scores on the test set, where T1 stands for top one and T2 stands for top two.

Form	MLP		LSTM		SVM	
	T1	T2	T1	T2	T1	T2
1	0.75	0.75	1.0	1.0	0.0	0.15
2	0.846	1.0	0.692	0.923	0.461	0.615
3	0.111	0.333	0.333	0.555	0.0	0.777
4	0.6	0.9	0.75	0.95	0.9	0.95
Overall	0.587	0.804	0.674	**0.869**	0.522	0.7519

**Table 8:** Confusion matrices for the considered patients; both LSTM (left) and MLP (right) networks are considered.

Predicted					Predicted				
Ground truth	4	0	0	0	Ground truth	3	1	0	0
	0	9	3	1		0	11	2	0
	0	1	3	5		0	4	1	4
	0	2	3	15		0	7	1	12

## Data Availability

The data used to support the findings of this study are available from the corresponding author upon request.
